# Quantitative Thin-Layer Chromatographic Method for Determination of Amantadine Hydrochloride

**Published:** 2008-06

**Authors:** Hassan F. Askal, Alaa S. Khedr, Ibrahim A. Darwish, Ramadan M. Mahmoud

**Affiliations:** 1 *Department of Pharmaceutical Analytical Chemistry, Faculty of Pharmacy, Assiut University, Egypt;*; 2 *Department of Pharmaceutical Analytical Chemistry, Faculty of Pharmacy, Al-Azhar University, Assiut, Egypt*

**Keywords:** amantadine hydrochloride, thin-layer chromatography, stability-indicating, pharmaceutical analysis

## Abstract

A simple and accurate thin-layer chromatographic (TLC) method for quantitative determination of amantadine hydrochloride (AMD) was developed and validated. The method employed TLC aluminum plates pre-coated with silica gel 60F-254 as a stationary phase. The solvent system used for development consisted of n-hexane-methanol-diethylamine (80: 40: 5, v/v/v). The separated spots were visualized as brown spots after spraying with modified Dragendorff’s reagent solution. Amantadine hydrochloride was subjected to accelerated stress conditions: boiling, acid and alkaline hydrolysis, oxidation, and irradiation with ultraviolet light. The drug was found to be stable under all the investigated stress conditions. The method was validated for linearity, limits of detection (LOD) and quantitation (LOQ), precision, robustness, selectivity and accuracy. The optical densities of the separated spots were found to be linear with the amount of AMD in the range of 5-40 µg/spot with good correlation coefficient (r=0.9994). The LOD and LOQ values were 0.72 and 2.38 µg/spot, respectively. Statistical analysis proved that the method is repeatable and accurate for the determination of AMD. The method, in terms of its sensitivity, accuracy, precision, and robustness met the International Conference of Harmonization/Federal Drug Administration regulatory requirements. The proposed TLC method was successfully applied for the determination of AMD in bulk and capsules with good accuracy and precision; the label claim percentages were 99.0 ± 1.0%. The results obtained by the proposed TLC method were comparable with those obtained by the official method. The proposed method is more advantageous than the previously published chromatographic methods as it involved the most simple chromatographic technique; TLC. In addition, method relies on the use of inexpensive equipment, a scanner and software, and not critical derivatizing reagent, thus maximizing the ability of laboratories worldwide to analyze samples of AMD.

## INTRODUCTION

**Figure 1:**
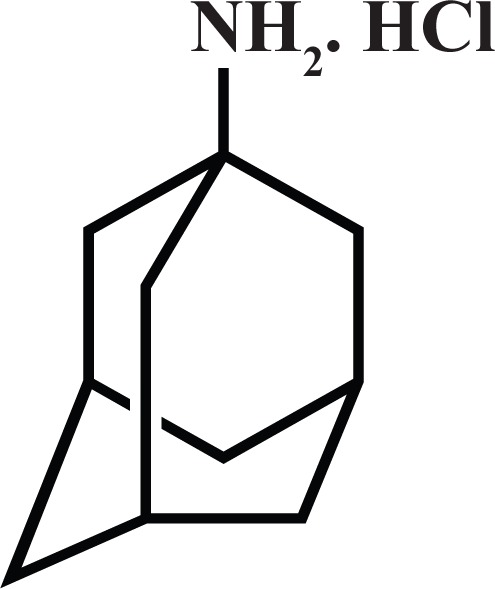
Amantadine HCl.

Amantadine (Fig 1); 1-aminoadamantine, is an antiviral agent used against infection with influenza type. A virus and to ameliorate symptoms when administered during the early stages of infection, as well as in the management of herpes zoster (1). It has mild antiparkinsonism activity and thus it has been used in the management of parkinsonism, mainly in early disease stage and when the symptoms are mild. Amantadine is usually given by mouth as the hydrochloride salt (2).

The analytical methods reported for AMD include high-performance liquid chromatography (3-6), gas chromatography (7, 8), capillary electrophoresis (9), potentiometry (10), and fluorimetry (11). Few spectrophotometric methods (12-14) have been reported for their determination. This is attributed to the absence of chromophores and/or auxochromes in the amantadine molecule. Thus, it shows no distinct absorption in the ultraviolet region above 200 nm, and direct UV spectrophotometry is not useful for its determination.

Thin-layer chromatography (TLC) is a routine analytical technique for the separation and identification of drugs. Its simplicity (require less sophisticated apparatus), low cost, need for minimum sample clean up, allows this type of analysis to be performed in remote areas. The TLC methods that have been described for AMD (15, 16) were devoted to its qualitative identification, relied on expensive densitometers, and/or do not indicate stability. The present study describes the development of a new alternative quantitative and not expensive stability-indicating TLC method for determination of AMD in bulk and capsule forms.

## EXPERIMENTAL

### Apparatus

The UVP scanner and software (Gel Works 1D Advanced Version 3.01) were obtained from Ultra Violet products Ltd. (Cambridge, UK). A test tube atomizer (12 ml) from Desaga GmbH (Wiesloch, Germany) was connected to a positive-pressure outlet valve of a membrane pump (Cole-Parmer, Chicago, USA). A thin-layer chromatographic spotting syringe (25 µl) was obtained from Hamilton (LKB, Bromma, Sweden). A TLC tank (standard type) and minivials lined with a silylated tetrafluoroethylene cap (1 ml) were products of Alltech (Mallinckrodt Chemical Works, New York, USA). Hot air oven (WTB binder 7200 Tuttingen, Schwa Bach, Germany), an UV lamp (Vilber lournate, Marne-lavallee Cedex, France), and a laboratory-made heating block were used.

### Materials

Thin layer chromatography aluminum plates (20 × 20 cm, 0.25 mm layer thickness) precoated with silica gel 60F-254 was obtained from Merck (Darmstadt, Germany). Nylon sample spraying filtration discs (0.45 μm) were obtained from (Riedel-De-Haen AG, Germany). Potassium iodide (El-Nasr Pharmaceutical Chemical Co., Abo-Zaabal, Egypt), bismuth subnitrate (Sigma-Aldrich Co. Ltd., Gillingham-Dorset, Germany), and sodium nitrite (Misr Co. for Pharmaceutical Industries, Cairo, Egypt) were used. Amantadine hydrochloride reference standard was obtained from Rameda Co. for Pharmaceutical Industries & Diagnostic Reagents (6^th^ October City, Cairo, Egypt) and used as received. Adamine capsules (Rameda Co. for Pharmaceutical Industries & Diagnostic Reagents, 6^th^ October, Cairo, Egypt) are labeled to contain 100 mg of adamantine HCl per capsule. HPLC-grade solvents, and other chemicals used throughout this study were of analytical grade.

### Preparation of standard solution

An accurately weighed amount (1 g) of AMD was transferred into a 10 ml calibrated flask and dissolved in about 5 ml of methanol. The resulting solution was completed to the mark with the same solvent to provide a stock standard solution containing 100 mg/ml. A measured volume (0.6 ml) of the stock solution was diluted to 10-ml to give 6 mg/ml as a working solution. Different volumes of this stock solution were transferred into a 10 ml calibrated flask and diluted to 10 ml with methanol to obtain the working standard solutions of concentrations suitable for analysis. For analysis 5 µl was applied onto the TLC plate.

### Preparation of capsule samples

The contents of 20 capsules were mixed, and accurately weighed amount of the contents equivalent to 100 mg of AMD was transferred into a 10 ml volumetric flask. Four milliliters of methanol were added and the contents of the flask were shaken for approximately 2 min. The solution was then diluted to the mark with the same solvent. The content of the flask was filtered through 0.45 µm filtration discs, and 6 ml of the filtrate was diluted to 10 ml to give a concentration of 6 mg/ml. For analysis, 5 µl was applied onto the TLC plate.

### Preparation of detection reagent

Forty grams of potassium iodide were as dissolved in 100 ml of distilled water (solution A). A 850 mg of bismuth subnitrate was dissolved in 50 ml of 20% (v/v) acetic acid (solution B). Immediately before use, a mixture of 10 ml of solution A, 10 ml of solution B, and 20 ml of glacial acetic acid were mixed and diluted with distilled water to 100 ml (Dragendorff's, reagent, solution C). Sodium nitrite solution (5%, w/v) was prepared by dissolving five grams of sodium nitrite in 100 ml of distilled water (solution D).

### Methods and procedures

**Sample loading.** The samples were applied to the marked start edge of the TLC plate at (1.5 cm height from lower edge of the plate) using the specified TLC-Hamilton glass syringe. The sample volumes for all experiments were 5 µl. The plate was then allowed to be air-dried for 10 min before its transferring to the TLC tank for the development.

**Chromatogram development.** The mobile system used was n-hexane-methanol-diethylamine (80:40:5, v/v/v). Fifty milliliters of the mobile system was poured into the TLC tank. The tank was then covered with a lined lid and pre-saturated with the mobile system vapor for at least 30 min at room temperature (25 ± 5°C) before use. The sample-loaded TLC plate transferred to the TLC tank the TLC plate was then developed for not less than a 15 cm migration distance of the solvent from the start line (about 30 min). The developed TLC plate was air-dried for about 10 min.

**Visualization of spots.** Spraying of the detection reagent was achieved by using double nozzle sprayer connected with the positive pressure outlet valve of a membrane pump. The distance between sprayer and the plate was about 30 cm. The plate was sprayed with Dragendorff’s reagent (approximately 8 ml was consumed), and the plate was air-dried for 10 min. The plate was then sprayed with 5% (w/v) sodium nitrite solution. The entire plate was turned brown because of the generation of iodine. The plate was allowed to air dry for at least 20 min at room temperature (25 ± 5°C). During this time, fading of the background occurs, and brown spots with a light yellow background persisted on the plate.

**Forced degradation**

- **Boiling:** One milliliter of AMD solution (200 mg/ml) was transferred to minivials. The vials were capped and inserted in to the heating block and heated at 100°C for 30 min. The vials were cooled before opening, and a volume of 5 µl (1 mg sample) was applied onto the TLC plate. Five µl of standard AMD solution (3 mg/ml) were applied.
- **Acid and alkali hydrolysis:** Aliquot of 1 ml of AMD solution (600 mg/ml) was transferred to minivial. The solution was mixed with 1 ml of 1 N hydrochloric acid, or 1 N sodium hydroxide. The prepared solutions were heated at 60 °C for 30 min with intermittent shaking. The samples were cooled to room temperature (25 ± 5°C), neutralized with an amount of acid or base equivalent to that of the previously added. From the resulting neutral solution (200 mg/ml), 5 µl (1 mg) was applied onto the TLC plate. Five µl of standard AMD solution (3 mg/ml) were applied.
- **Oxidation:** One milliliter of AMD solution (400 mg/ml) was transferred to minivials. The vial content was then mixed with 1 ml of 10% hydrogen peroxide solution, and heated at 60°C for 30 min with intermittent shaking. A volume of 5 µl (1 mg) was applied onto the TLC plate. Five µl of standard AMD solution (3 mg/ml) were applied.
- **Irradiation with ultraviolet light:** A sample powder of AMD (200 mg) was exposed to UV light for 7 days. The vial contents were dissolved in 2 ml methanol. The solution was filtered with syringe filtration disk and diluted to 20 ml with water to obtain a claimed concentration of 5 mg/ml. Five µl of standard AMD solution (3 mg/ml) were applied.
- **Heating of capsules contents (incompatibility testing):** The contents of one capsule of AMD were transferred into minivial (two vials for each product). One of them was mixed with 10 µl of water while the other was leaved dry. The vials were capped tightly and allowed to stand in heating incubator at 60°C for 14 days. The vial contents were dissolved in methanol. The solutions were filtered with 0.45 µm filtration disc, and diluted to obtain a claimed concentration of 200 mg/ml. A volume of 5 µl (1 mg/spot) was applied onto the TLC plate. The applied concentration was higher than that of the standard samples used in generating the calibration curve (5-40 µg/spot) to assure the detection of any minor incompatibility products.

### Data processing and treatment

The TLC chromatogram, as an image, is captured by the scanner and the image is then loaded into the Gel Works software. In the software, the following operations are performed:

- The series of spots to be manipulated are selected as a lane by lane creation function. Once the lane is created, a chromatogram is generated. The generated chromatogram is a function of spot position with the corresponding optical density, represented as pixel intensity.
- The background of the TLC chromatogram, if any, is subtracted.
- After background subtraction, the signals of the chromatogram are assigned with numbers according to the sequence of the corresponding spots in the previously selected band.
- Quantity calibration is then performed by pre-assignment of the concentration of the active material of each spot. Once the authentic known concentrations are assigned, the calibration graph is automatically generated. The generated graph correlates the concentration with the corresponding signal intensity (represented as raw volume).

**Figure 2:**
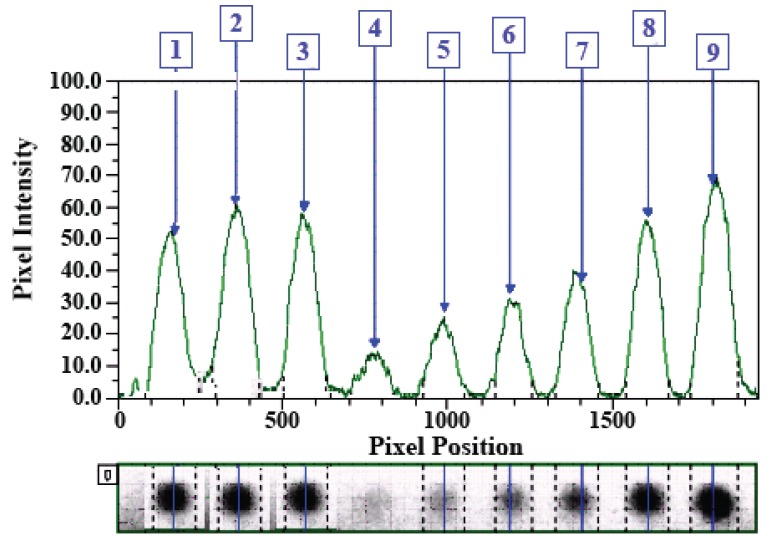
Analysis of AMD samples (spots 1-3) nominated to contain 30 μg/spot by one-point calibration curve of on-plate derivatized AMD. The concentrations of AMD were 5, 10, 15, 20, 30, and 40 μg/spot for spots 4-9, respectively.

Once the above-mentioned operations are performed the software provides a report for all operations. Figure 2 illustrates an example of a chromatogram generated for the analysis of three AMD samples by one-point calibration curve of on-plate derivatized AMD.

## RESULTS AND DISCUSSION

### Method development

Initial method development was conducted on pure drug using working standards solution. Actual chromatographic conditions were established after number of preliminary experiments involving selection of the proper mobile system and detection reagent. Different mobile systems were tested. The strategy for the selection of the proper mobile phase was the changing in the components and their ratios to attain R_f_ value of 0.5 ± 0.1. The proper mobile system consisted of n-hexane-methanol-diethylamine (80:40:5, v/v/v). This system gave compact spots for AMD (R_f_ values of 0.45). For detection of AMD on the plates, several reagents were tested; these reagents were ninhydrin, iodine, Marqui’s reagent, and Dragendorff’s reagent. Ninhydrin required heating at 120°C for 30 min. Iodine (as vapor) required long contact time for spot development and the colors of the spots were faded rapidly. Marqui’s reagent could not be applied conveniently by spraying because it contains the corrosive sulphuric acid and harmful formaldehyde. In comparison with these reagents, Dragendorff’s reagent is more convenient alternative as it reacted at room temperature, besides it is had a lower cost. The color of the spot developed by Dragendorff,s reagent was orange with faint orange background, however the colors contrast was not adequate for achieving good sensitivity. Therefore, the plate was sprayed with sodium nitrite solution (5%, w/v), which generates iodine from the previously applied Dragendorff,s reagent, when the plate was allowed to stand for some while, brown spots with light yellow background were developed.

### Method validation

**Linearity and limits of detection and quantitation.** Using the above-mentioned optimum mobile system, chromatogram development, and detection reagent, a three-point calibration graph correlating the optical density as pixels (expressed as raw volume) with the corresponding AMD concentration was constructed. Regression analysis for the results was carried out using the least-square method. The results revealed a good linear calibration fit in the range of 5-40 µg/spot. The calibration equation, generated automatically once the method of calculation was defined, was:

OD = -1.146 + 1.389 C    (r=0.9994)

where OD is the optical density (pixel intensity), C is the concentration of AMD, and r is the regression coefficient. The calibration curve was valid only for the calculation of concentration of AMD separated on the same plate.

The limits of detection (LOD) and limits of quantitation (LOQ) were determined by fitting the inter day standard deviations (SDs) calculated for each calibration curve. The intercept was then equal to SD0 (the estimated SD at a concentration of zero). LOD was then defined as 3SD0, and LOQ was defined as 10SD0. The LOD and LOQ values were 0.72 and 2.38 µg/spot, respectively.

**Figure 3:**
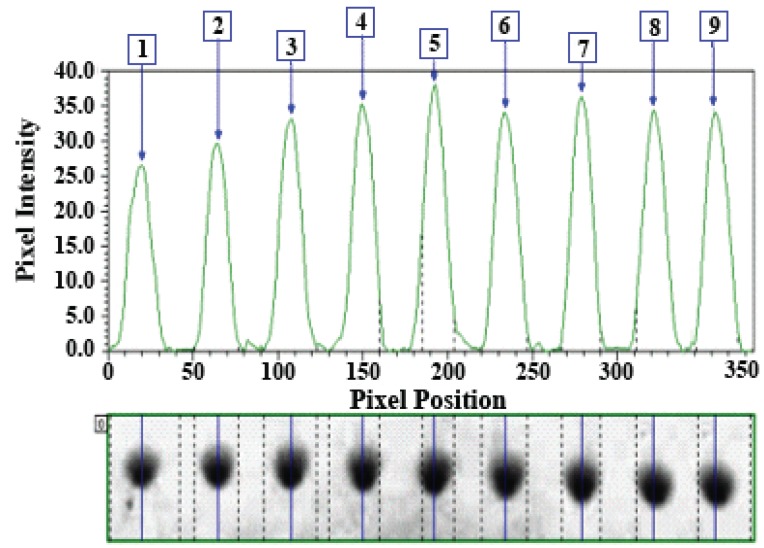
Replicate analysis of 25 μg/spot of AMD by TLC for testing of system suitability and method precision.

**System suitability, precision, and accuracy.** System suitability testing was performed on TLC plates using a standard AMD solution of 25 µg/spot. The precision of the proposed method was determined by replicate analysis of nine separate samples of the same solution; results are shown in Figure 3. The relative standard deviation (RSD) of the calculated R_f_ values was close to zero, and the RSD of the raw volumes (pixel intensities) were 4.73%. This good level of precision was suitable for quality control analysis of AMD. Accuracy was checked by analysis of AMD sample solutions at three concentration levels (10, 15, and 30 µg/spot), by calibration curve generated on the same TLC plate. The error did not exceed 0.41% (Table 1).

**Table 1:** Accuracy of the proposed TLC method for analysis of AMD

Nominated conc. (μg/spot)	Measured conc. (μg/spot)	RSD (%)	Deviation^a^ (%)

10	10.00 ± 0.01^b^	0.08	0.07
15	14.81 ± 0.25	0.25	0.08
30	29.99 ± 0.53	0.92	0.41

a Deviation (%) = (nominated conc. – mean measured conc.) ×100/nominated conc;

b Values are mean of three determinations ± SD.

**Robustness and ruggedness.** In order to measure the extent of the method robustness, the most critical parameters were interchanged while keeping the other parameters unchanged, and in parallel the chromatographic profile was observed and recorded. The chromatographic parameters were interchanged within the range of 1-10% of the optimum recommended conditions. The studied parameters were: the composition of the mobile phase, amount of spraying reagent, and color development time. The results indicated that the small change in the composition of the mobile phase did not significantly affect the R_f_ value of AMD.

Ruggedness The ruggedness of the method was evaluated by applying the analysis of AMD solutions using two different TLC plates from two different manufacturers; Merck and Fluka Chemie GmbH (Germany) of different layer thicknesses (0.25 and 0.20 mm). Both plates yielded the same resolution efficiency and R_f_ values; however, the thinner-layer plate required a longer time for the solvent to reach the marked front. A compact spots without tailing was observed in both cases. Awareness should be paid to the amount of samples delivered from the spotting syringe because this step expresses the material concentration. A calibrated spotting device could be useful to avoid sample volume variation. However, the loaded volume area variation did not make any difference in the results.

**Sample solution stability.** According to the experimental procedure described above, the preparation and loading of 20 samples on the plate takes approximately 30 min, which is enough time to allow for much material degradation, if it is degradable. Therefore, it was necessary to investigate the solution stability during the analysis time. The results revealed that no degradation occurred during run time, and the solution was stable for at least 2 hours.

### Stability-indicating study

**Figure 4:**
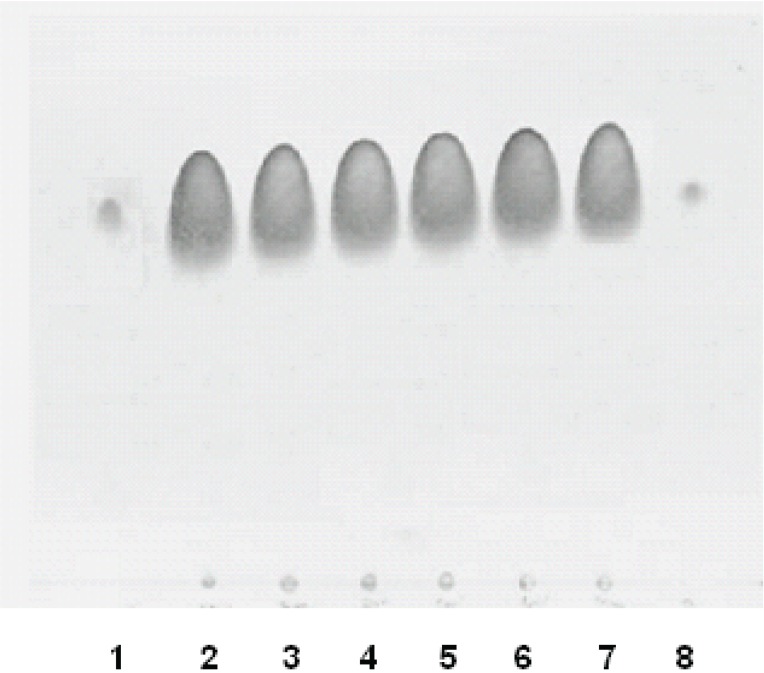
Stress testing of AMD. Lanes 1 and 8 are the AMD standard solution. Lanes from 2 to 6 are samples that have been subjected to alkali hydrolysis, oxidation, acid hydrolysis, ultraviolet irradiation, and boiling, respectively. Lane 7 is the capsule content subjected to moisture.

The International Conference of Harmonization (ICH) guideline entitled stability testing of drug substances and products requires the stress testing to be carried out to elucidate the inherent stability characteristics of the active substance, and provide a rapid identification of differences that might result from changes in the manufacturing processes or the source sample (17). Susceptibility to oxidation, hydrolytic, and photolytic stability are the required tests. An ideal stability-indicating method is one that quantifies the standard drug alone and also resolves its degradation products. A relatively high concentration (1 mg) of AMD was used to assure the detection of minor degradation products (18). As described in the experimental section, different stress conditions were applied: boiling, acid, base hydrolysis, oxidation, and irradiation with UV light. From this investigation, it was clear that AMD was stable under all the stress conditions, as the chromatogram did not show any additional spots for degradation spots (Fig. 4).

The compatibility of AMD with the exipients used was also studied in the presence and absence of moisture. The stress testing results revealed that AMD was compatible with the combined exipients, whereas no more degradation products were observed when the stress testing experiments carried out on AMD-containing capsules (Fig. 4).

### Application to the analysis of capsules

It is evident from the results obtained previously that the proposed method gave satisfactory results with the analysis of AMD in bulk. Thus adamine® capsules were subjected to the analysis for their contents of AMD by the proposed method as well as the official method (19). The label claim percentage was 98.99 ± 0.98%. These results were compared with those obtained from the official method by statistical analysis with respect to the accuracy (t-test) and precision (F-test). No significant differences were found between the calculated (t- and F values were 1.75 and 3.85, respectively) and theoretical values of both the proposed and the reported methods at 95% confidence level; the theoretical values of t- and F- (at n=3) were 3.18 and 9.01, respectively. This indicated similar accuracy and precision in the analysis of AMD in capsules form.

## CONCLUSIONS

The present study represents the development and validation of a simple, accurate, and sensitive TLC method for quantitative determination of AMD. From an economics point of view, the method used the most simple and cost effective chromatographic technique; TLC. The method relies on the use of inexpensive equipment; scanner and software. Additionally, all of the analytical reagents are inexpensive, and are available in any analytical laboratory. The proposed method could be recommended for routine use in quality control laboratories.
